# *Listeria* Placental Infection

**DOI:** 10.1128/mBio.00949-17

**Published:** 2017-06-27

**Authors:** José A. Vázquez-Boland, Emilia Krypotou, Mariela Scortti

**Affiliations:** Division of Infection and Immunity, The Roslin Institute and Edinburgh Medical School (Biomedical Sciences), University of Edinburgh, Edinburgh, United Kingdom

**Keywords:** *Listeria* miscarriage, *Listeria monocytogenes*, maternofetal listeriosis, placental infection

## Abstract

The Gram-positive facultative intracellular bacterium *Listeria monocytogenes* is the causative agent of listeriosis, a severe food-borne infection. Pregnant women are at risk of contracting listeriosis, which can potentially lead to miscarriage, stillbirth, preterm birth, and congenital neonatal infections. While other systemic bacterial infections may result in adverse pregnancy outcomes at comparable frequencies, *L. monocytogenes* has particular notoriety because fetal complications largely occur in the absence of overt illness in the mother, delaying medical intervention. Here, we briefly review the pathophysiology and mechanisms of maternofetal listeriosis, discussed in light of a recent *mBio* report on *Listeria* transplacental infection in a nonhuman primate model.

## COMMENTARY

Although many human systemic bacterial infections may result in miscarriage or stillbirth, unlike *Listeria monocytogenes*, the causative agents are not considered primarily abortifacient. For example, recent data from the United Kingdom show that of a cohort of 75 pregnant women with invasive *Haemophilus influenzae* infection, 63% had fetal loss (1). In comparison, with a similar incidence, only 40% of pregnancy-associated listeriosis cases resulted in spontaneous abortion, stillbirth, or neonatal death in the same geographic area (2). Other examples include *Brucella*, *Campylobacter*, *Coxiella*, or *Salmonella*, to name a few; while not recognized as part of the TORCH (toxoplasmosis, other infections, rubella, cytomegalovirus, and herpes simplex virus) group, all these pathogens may lead to adverse fetal outcome in pregnant women (3–6) and are also well-known causes of septic abortion in farm animals (7). It has been suggested that essentially any invasive bacteria with the ability to survive within host cells can potentially colonize the placenta and fetus via the hematogenous route (8). What then makes *L. monocytogenes* stand out as a notorious miscarriage-associated microbe? One could argue that this is largely due to perception, owing to the inapparent or mild clinical course of maternal infection before the onset of obstetric signs. This is in contrast to other systemic infections, where fetal complications are mainly regarded as secondary to the mother’s illness.

The absence of obvious outward manifestations preceding listerial miscarriage has been experimentally observed in a study with intragastrically (i.g.) infected cynomolgus macaques recently published by Wolfe et al. in *mBio* (9). Similar findings were previously reported in rhesus macaques (10, 11), confirming that the lack of specific prodromal signs appears to be characteristic of maternofetal listeriosis. With a similar hemochorial, villous, discoidal placenta and reproductive cycle, nonhuman primates offer a suitable model to study human reproduction and related disorders (12, 13). Macaques are naturally susceptible to *L. monocytogenes* infection, with clinical outcomes in pregnant monkeys mirroring those in human maternofetal listeriosis. This includes miscarriage or stillbirth in late pregnancy (14) (in pregnant women mostly during late second/third trimester [2, 15]).

### Timing of listerial miscarriage.

The study by Wolfe et al. (9) is interesting in that the monkeys were inoculated in early gestation (single i.g. dose of 10^7^ CFU between gestation days [GD] 36 to 46, term is GD 165), and all four exposed animals miscarried. In contrast, in an earlier study, Smith et al. (11) observed only six stillbirths among 23 late pregnancies following i.g. exposure of macaques at around GD 110. Wolfe et al. concluded that the macaque’s maternal-fetal interface may be particularly sensitive to *Listeria* infection in early pregnancy and that there might be a greater, unrecognized risk of listerial miscarriage in the first trimester of gestation (9).

However, careful analysis of the existing data for macaques indicates that the suggested increased susceptibility in early pregnancy (9) may be more apparent than real, due to the high experimental infection dose used. In late pregnancy monkeys, Smith et al. (11) observed 16.6% stillbirths after i.g. exposure to 10^3^ to 10^4^ *L. monocytogenes* CFU, 28.6% stillbirths when using 10^5^ to 10^6^ CFU, whereas the only monkey administered 10^7^ CFU, i.e., the same dose as Wolfe et al. (9) used, delivered a stillborn fetus. No adverse fetal outcomes were observed with a 10^2^ dose. For a given single-dose *L. monocytogenes* inoculum, a higher rate of blood-borne colonization per tissue mass is to be expected for a smaller uteroplacental unit than for a significantly more-developed one. An overwhelming placental infection in Wolfe et al. is supported by the acute course and short incubation period before fetal demise, only 7 to 13 days postinoculation (9) compared to an average of 47 days until stillbirth in the Smith et al. study (11). The latter is more in keeping with the typical incubation period of maternofetal listeriosis in humans (16), generally associated with lower levels of exposure to *L. monocytogenes* according to outbreak data (5 × 10^4^ CFU/g in the incriminated food [17]; 1.9 × 10^6^ estimated dose for 50% listerial perinatal morbidity [18]).

**FIG 1:**
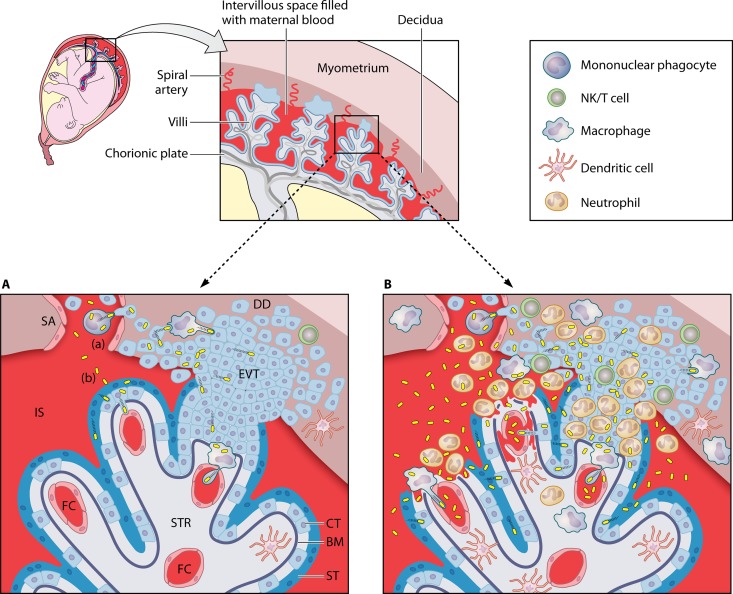
*Listeria* transplacental infection. (Top) A pregnant human uterus and schematic of the maternal-fetal interface. Anchoring and floating villi are represented. (Bottom) Magnified diagrams of an anchoring villus and its main structural and cellular components illustrating two transplacental invasion scenarios. (A) Low-level infection with stealthy, mainly cell-to-cell spread-based transplacental dissemination; (B) acute infection with strong inflammatory response, major disruption of the integrity of the placental barrier with degeneration of syncytiotrophoblast, and significant hematogenous dissemination. The diagram in panel A shows the uterine decidua (DD), the end of a spiral artery (SA) from which maternal blood flows into the intervillous space (IS), and a chorionic villous tree lined by the syncytiotrophoblast (ST) with underlying progenitor cytotrophoblast (CT) cells and a basement membrane (BM). CT cells penetrate into the decidua to anchor the villi in the uterus and invade the maternal arteries to allow blood extravasation into the IS. The villous stroma (STR) contains fetal capillaries (FC) that are located closer to the villous surface as pregnancy advances. Panel A illustrates the two main hematogenous placental invasion pathways: (a) actin-based cell-to-cell spread from infected phagocytes that traffic from primary infectious foci in maternal organs to the placenta (Fig. 2); (b) invasion of the trophoblast by free blood-borne listeriae. Bacteria are shown in yellow (not drawn to scale).

A number of factors may explain the predominant presentation of maternofetal listeriosis in late pregnancy. For example, the human placenta is thought to become truly hemochorial only in the second trimester (19). This late shift in the nature of the maternofetal interface appears to be unique to great apes. In the macaque, intervillous maternal circulation is established as early as 3 weeks after conception (12, 13), which together with an earlier invasion of spiral arteries by the extravillous trophoblast (EVT) (12) (the primary placental cellular target of *L. monocytogenes*; see below) (Fig. 1) may contribute to explain the exquisite susceptibility noted by Wolfe et al. (9). The importance of the placental configuration is unclear, however, because listerial abortion in ruminants is also predominantly observed in late pregnancy (7, 20) despite having a different type of placentation (epitheliochorial, with the maternal and fetal circulations physically separated by six tissue layers) (13).

**FIG 2:**
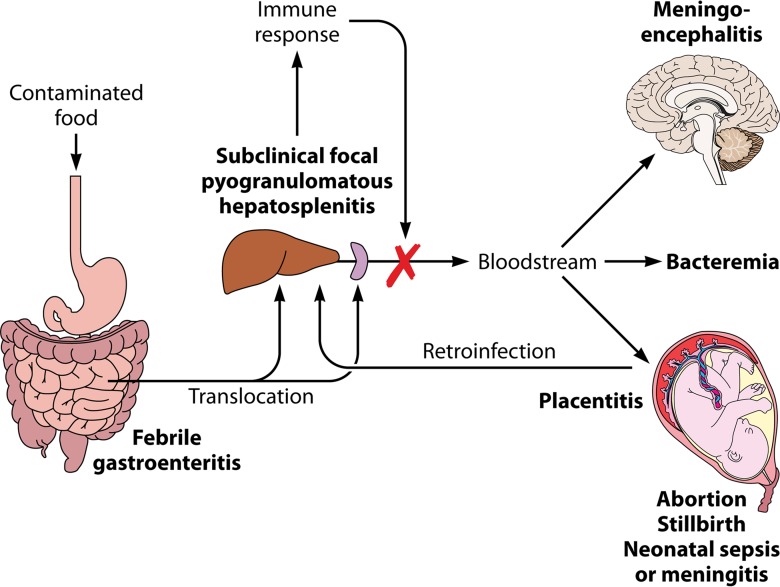
Pathophysiology of food-borne listeriosis. *L. monocytogenes* bacteria cross the epithelial barrier of the intestine, translocate to the mesenteric lymph nodes, and reach their primary target organs, i.e., liver and spleen. There they establish infectious foci that in an immunocompetent individual are efficiently cleared by cell-mediated immunity. In adult people with no predisposing conditions, the process is largely subclinical. In this population, exposure to larger infective doses may cause febrile gastroenteritis and, in rare cases, invasive disease. In immunocompromised adults and elderly people who are unable to mount an efficient T-cell-mediated immune response, the primary infectious foci are inadequately resolved and *Listeria* bacteria may be released to the bloodstream. This results in febrile bacteremia and, eventually, invasive infection of the brain. In pregnant women, *L. monocytogenes* colonizes the uterus in addition to the liver and spleen. While the infection is controlled in the latter organs, the placental immune tolerance mechanisms provide a permissive niche for the proliferation of *L. monocytogenes*. Bacteria from the placental reservoir released to the bloodstream may reinfect the mother’s liver and spleen, contributing to infection maintenance and amplification (21). Transplacental dissemination to the fetus results in abortion, stillbirth, or neonatal sepsis. A late-onset congenital form is also observed in neonates, often accompanied by meningitis. Based on an original depiction in reference 38.

Other possible explanations include the following: (i) predominance of low-level infections requiring a protracted incubation period, where the placenta itself may become a reservoir for maternal reinfection and amplification of the bacterial load (21); (ii) progressive increase in uterine blood flow (and hence exposure of the placenta to blood-borne listeriae) (Fig. 2) relative to the overall cardiac output; (iii) possible exacerbation of critical immune tolerance mechanisms at the maternal-fetal interface in late gestation; (iv) physiological burden of advanced pregnancy, as suggested by the observation that pregnant women carrying multiple fetuses are at greater risk of listeriosis (22); and (v) fetal death during early gestation being more likely to go unreported or remaining etiologically undiagnosed.

### Mechanism of placental colonization and dissemination to the fetus.

The high-dose infection used by Wolfe et al. caused an extensive neutrophilic inflammatory response with disruption of the maternal-fetal barrier of the macaques (9). Vasculitis, thrombosis, and necrosis of the decidual spiral arteries and the presence of bacteria in the intervillous maternal circulation, villous capillaries, and umbilical cord were consistently observed (Fig. 1B). While inflammation-mediated hematogenous transmission to the fetus may prevail in an acute or advanced placental infection, the importance of this dissemination pathway at the initial stages of the process or in cases of low-level infections is less clear. *L. monocytogenes* is an actively invasive pathogen endowed with a stealthy actin-based cell-to-cell spreading mechanism that bypasses the dependency on cell/tissue damage for successful dissemination while allowing escape from immune control (23) (Fig. 1A). The key importance of this cell-to-cell spread mechanism in transplacental colonization has been demonstrated *in vivo* in nonprimate animal models using *L. monocytogenes* mutants lacking the virulence factor responsible for it, the actin-polymerizing protein ActA (24, 25).

The primary hematogenous entry route to the placenta (Fig. 2) is more controversial. Studies of guinea pigs, gerbils, and mice show a contributing yet dispensable role for the listerial invasins InlA and InlB (required for internalization into nonphagocytic host cells but not for uptake by professional phagocytes) in placental and fetal colonization (24, 26, 27). Thus, direct invasion by extracellular bacteria seems less likely to be the main mechanism than cell-to-cell spread from infected maternal phagocytes trafficking to the placenta (21) (Fig. 1A). The predominance of one mechanism over the other may critically depend on the infectious dose and degree of infection of the primary target organs (liver and spleen) after listerial intestinal translocation (Fig. 2).

### Placental tropism?

Wolfe et al. (9) observed substantially larger bacterial loads in the decidua and placenta, umbilical cord, amniotic fluid, and fetal tissues (>10^7^ to 10^8^ CFU) compared to the maternal nonreproductive tissue (liver, spleen, and lymph node; <10^5^ CFU). This was interpreted as a demonstration of the tropism of *L. monocytogenes* for the gravid uterus (9). Alternatively, it may simply reflect the relative permissiveness of the placenta to listerial infection compared to other (immunologically) more restrictive maternal tissues and organs, i.e., “passive tropism.” The fetally derived cytotrophoblast which forms the maternal-fetal interface, particularly the invading EVT cells that penetrate into the decidua and the maternal spiral arteries from the anchoring villi, appear to be the primary target for *L. monocytogenes* placental colonization (24, 25, 28, 29) (Fig. 1). Trophoblast cells lack class I HLA-A and HLA-B antigens and class II antigens while expressing nonclassical HLA class I molecules, dampening allorecognition by uterine NK cells and T cells (30–32). This and other placental immune tolerance mechanisms prevent the rejection of the semiallogenic fetus but at the same time may provide a protected sanctuary for the proliferation of intracellular pathogens like *L. monocytogenes*, ultimately depending on T-cell-mediated immunity for clearance (33). Consistent with the rare cooccurrence of central nervous system (CNS) involvement in maternofetal listeriosis (0.01%) (15), neurological signs were never observed in experimentally infected pregnant macaques (9–11) despite listerial CNS infection having a shorter incubation period (1 to 14 days) (16). In an immunocompetent pregnant mother, this may reflect competition between a permissive placenta and a less permissive blood-brain barrier in allowing the establishment of limited numbers of circulating listeriae (Fig. 2). The fact that the placenta and fetus are also preferential infection sites for *L. monocytogenes* in ruminants despite the structurally and cellularly distinct interhemal barrier argues against the implication of specific targeting mechanisms (unless involving a conserved, promiscuous host receptor); it suggests rather that placental invasion depends on general intrinsic features of the *Listeria* host-pathogen interaction (such as, e.g., cell-to-cell spread in an immunologically relatively permissive environment).

The above does not exclude the existence of *L. monocytogenes* determinants facilitating establishment and proliferation in the maternal reproductive tract and placenta. According to recent data in guinea pigs, a listerial protein of the internalin family, InlP, which is generally conserved in *L. monocytogenes*, appears to specifically aid placental colonization (34). Virulence heterogeneity among *L. monocytogenes* isolates is well documented, and specific “hypervirulent” clonal complexes have been epidemiologically and experimentally associated with invasive (placental and CNS indistinctly) listeriosis (35). Anecdotal evidence hints at the possibility that particular strains might be more prone to cause maternofetal infections versus other clinical presentations. Thus, some listeriosis outbreaks, associated with a specific *L. monocytogenes* strain, have an unusually high frequency of maternal-perinatal cases (36, 37). In ruminants, maternofetal and CNS forms of the disease seldom occur simultaneously in the same herd outbreak (20, 38). Interestingly, considered together, the studies by Wolfe et al. (9) and Smith et al. (10, 11) show that two strains associated with maternofetal listeriosis cases in humans or primates consistently caused miscarriage in experimentally infected macaques with an i.g. dose of 10^7^, while two other strains involved in human outbreaks with a low frequency of maternofetal cases did not. Specifically, a *L. monocytogenes* strain (ScottA) from a listeriosis outbreak with few pregnancy-related cases (7 of 42) (39) caused stillbirth at a dose of 10^10^ (1/1) but not 10^8^ (0/1) unless as part of a mix with isolates associated with maternofetal infections (2/2) (10). Although based on very limited evidence and far from conclusive, these data show a trend that warrants further investigation.

As briefly outlined here, many areas still remain obscure in our understanding of maternofetal listeriosis. Key points for attention include the impact of the bacterial dose on placental infection dynamics, the pathophysiology and determinants of transplacental invasion, and the potential involvement of tropism factors. Also requiring clarification is the role of early immune signaling events prior to transplacental colonization as a precipitating factor in fetal demise (40). Experiments in relevant animal models, including macaques closely replicating the human system, should prove invaluable for illuminating the detailed mechanisms of placental listeriosis and other aspects of *Listeria* pathogenesis.
